# Supplementary material for: A Comparative Study of the Efficacy of an Intervention with a Nutritional Supplement for Patients with Chronic Kidney Disease: A Randomized Trial
Source: J Clin Med. 2022 Mar 16;11(6):1647. doi: 10.3390/jcm11061647 (PMC8951226; doi:10.3390/jcm11061647)
Supplement: Supplementary file 1 [file jcm-11-01647-s001.zip › jcm-1611711-supplementary.pdf]

Supplementary Table S1: Nutritional values of the oral supplement FontActiv® renal

**Nutritional values, *unit***	**Per 100 mL**
Energy, *kcal*	197
Energy, *kJ*	824
Total fat, *g*	9.5
Saturated fat, *g*	1.5
Monounsaturated fat, *g*	5.5
Polyunsaturated fat, *g*	2.5
Carbohydrates, *g*	19
Sugars, *g*	0.6
Lactose, *g*	<0.5
Fiber, *g*	1.7
Protein, *g*	8.0
Salt, *g*	0.13
Sodium, *mg*	51
Potassium, *mg*	29
Chlorine, *mg*	32
Calcium, *mg*	98
Phosphorous, *mg*	63
Magnesium, *mg*	2.10
Iron, *mg*	1.4
Zinc, *mg*	1.9
Copper, *µg*	250
Manganese, *mg*	0.4
Fluor, *mg*	0.02
Selenium, *µg*	8.5
Chromium, *µg*	4.2
Molybdenum, *µg*	12
Iodine, *µg*	40
Vitamin A, *µg*	118
Vitamin D, *µg*	3.2
Vitamin E, *mg*	2.5
Vitamin K, *µg*	20
Vitamin C, *mg*	14
Vitamin B1 (thiamine), *mg*	0.2
Vitamin B2 (riboflavin), *mg*	0.45
Vitamin B3 (niacin), *mg*	1.7
Vitamin B6, *mg*	0.46
Folic acid, *µg*	60
Vitamin B12, *µg*	0.85
Biotin, *µg*	6
Vitamin B5 (pantothenic acid), *mg*	1.2
